# Talking about cancer: Patient responses to raising awareness of oral cancer in primary dental care

**DOI:** 10.1111/cdoe.12783

**Published:** 2022-08-14

**Authors:** Suzanne E. Scott, Geanina Bruj, Shahryar Beheshti, Ruth Evans, Oluwatunmise Awojobi

**Affiliations:** ^1^ Wolfson Institute of Population Health Queen Mary University of London London UK; ^2^ Faculty of Dentistry, Oral & Craniofacial Sciences King's College London London UK; ^3^ Guy's & St Thomas' NHS Foundation Trust London UK

**Keywords:** communication, early diagnosis, fidelity, observation, oral cancer

## Abstract

**Objectives:**

Dentists and other members of the dental team could raise awareness by talking about oral cancer during routine dental check‐ups. A communication guide has been developed to facilitate this. However, it has been suggested that discussions about oral cancer may raise patients' anxiety and this has been documented by dentists as a barrier to having these conversations. The current research aimed to investigate implementation of the communication guide and its impact on the dental patient.

**Methods:**

A consecutive‐case sample of adult dental patients attending primary dental care for a routine NHS check‐up at one dental practice were invited to take part in the study via letter prior to their appointment. Consultations of participating patients (*n* = 77) were audio‐recorded. Before and after their appointment, patients were asked to rate their current anxiety via the six‐item version of Spielberger's State‐Trait Anxiety Inventory. Audio recordings of each consultation were reviewed by two raters to determine the extent to which the dentist covered the topics recommended in the communication guide.

**Results:**

The dentist informed all patients that they were being checked for oral cancer, spoke about signs and symptoms, and discussed risk factors. However, they rarely recommended where help should be sought or addressed barriers to seeking help. Discussions took an average of 95 s. The extent to which oral cancer was discussed did not correlate with patients' post‐appointment anxiety. Patients made positive or neutral responses to the discussions. The few questions that were asked were easily addressed.

**Conclusions:**

As findings are based on one dentist working at one practice, generalization of these results should be cautious. The study indicated that using an evidence‐based guide to talk about oral cancer did not appear to raise patients' anxiety in this practice population. This could help to increase awareness of oral cancer in the endeavour to facilitate early cancer diagnosis.

## 1 INTRODUCTION

In the UK the number of people diagnosed with oral cancer is increasing. Between 2004–2006 and 2014–2016 incidence rates have increased by 22%.^1^ Patients with a tumour detected at Stage I have an 85% five‐year survival, compared with 10% for those diagnosed at stage IV.^2^ Identification and treatment at an advanced stage is often associated with significant health‐related impairments including speech, eating and disfigurement, all of which have a profound impact on the patient's quality of life.^3^ In England, over 50% of cases of oral cancer are diagnosed at an advanced stage.^4^ One explanation for the high occurrence of advanced stage oral cancer is delays in seeking help for symptoms.^5^ Population‐based data indicates that in England, individuals subsequently diagnosed with oral or oropharyngeal cancer have the longest patient intervals (time from symptom onset to first consultation with a healthcare professional) compared with 27 other cancers.^6^ Approximately 30% of patients wait more than 3 months before consulting a healthcare professional about signs of oral cancer.^5^ Misinterpretation of symptoms, low cancer awareness, competing priorities and low perceived ability to access health care have been found to be key explanations for delayed presentation of oral cancer.^5^, ^7^, ^8^ The general population possess poor knowledge about oral cancer, and some key points (such as signs, symptoms and risk factors) are not well understood.^9^, ^10^, ^11^ In a recent survey, over 50% of participants could not identify the signs and symptoms of oral cancer, 63% identified smoking cigarettes as a cause of oral cancer but only 35% identified alcohol as a risk factor.^9^ People at higher risk of oral cancer, such as smokers, may have lower awareness than that of non‐smokers.^12^ It is therefore essential to develop successful methods to aid early presentation by raising awareness of oral cancer, helping patients evaluate oral symptoms and increasing their ability to receive help for symptoms in a timely manner.^13^, ^14^, ^15^

Members of the dental team could have a key role in raising awareness of oral cancer.^16^ For instance, dentists and dental hygienists screen patients for signs of oral cancer and when doing so could also talk to patients to raise awareness.^17^, ^18^, ^19^, ^20^ Yet, a household survey of a nationally representative sample of 3384 adults^11^ found that only 7.1% of those surveyed reported that their dentist or doctor had spoken to them about oral cancer. Similarly, a cross‐sectional study of 184 adult dental patients indicated that a low proportion of participants were aware of being screened for oral cancer by their current dentist (14%) or ever (12%).^20^ Data from the U.K. and U.S. indicate that dentists are often reluctant to tell patients they are performing an oral mucosal examination, and often avoid using the word ‘cancer’ altogether as they are concerned about alarming patients and do not believe patients would be interested in this health advice.^19^, ^20^, ^21^ In contrast, research with patients indicates dental patients (including that those at risk of developing oral cancer) are in favour of discussing oral cancer with their dentist.^21^ A dental patient survey indicated 92% wanted their dentist to inform them that they are being screened for oral cancer and 97% wanted to be supported to reduce their risk of developing the disease.^20^

**FIGURE 1:**
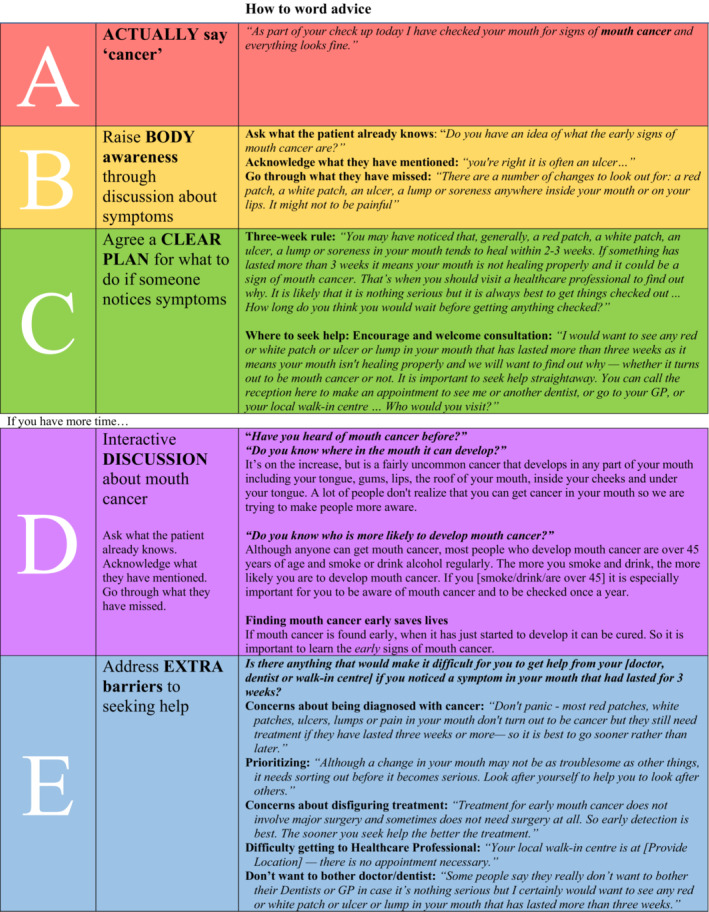
Communication guide: the ABC(DE) of encouraging early diagnosis of mouth cancer in general dental practice.

Given dentists' reluctance to raise the topic of oral cancer, finding ways to help the dental team talk to patients about oral cancer during a routine examination is important.^20^ An evidence‐based communication guide, the ‘*ABC(DE) of encouraging early diagnosis of mouth cancer in the general dental practice*’ (Figure 1) offers clear guidance to equip dentists to communicate effectively about oral cancer with their patients.^22^, ^23^ The guide was developed and tested through consultation with key stakeholders including adult dental and medical patients, General Dental Practitioners, General Practitioners, specialists in Oral Medicine, Dental Public Health Practitioners and Health Psychologists.^18^, ^20^, ^24^ The content of this guide is based on theory (e.g. Leventhal's Common Sense Model of Self‐regulation, Bandura's Social Cognitive Theory, see Model of Pathways to Treatment^25^) and evidence about the reasons why people delay seeking help from a healthcare professional after noticing symptoms of mouth cancer,^5^, ^13^, ^14^ alongside principles of effective communication as specified in the Calgary Cambridge Communication Guide.^26^ It includes key messages in an easy‐to‐follow format for an interactive discussion about symptoms, the importance of early detection, and when and where to seek help should symptoms occur. More than just providing information to the patient, it ensures a patient‐centred brief discussion to allow for personally relevant information to be shared, increasing the likelihood that the patient will engage with the discussion and increase their awareness of oral cancer. The guide emphasizes the ‘three‐week rule’ to help patients evaluate the need for care and encourages the dentist to negotiate a personalized action plan of where to seek help should symptoms occur in the future, taking into account any perceived barriers to accessing care.

Evaluation of training in the use of this guide found dentists reported fewer perceived barriers to communicating about oral cancer and became more confident to talk about oral cancer with patients after attending the training session.^23^ However, there are questions surrounding implementation of the oral cancer communication guide into routine dental practice (i.e. how the guide is used or adapted in everyday practice). Even after training, some dentists still seemed concerned about raising patient anxiety; therefore, it is vital to obtain data on patients' responses to talking about oral cancer in the dental setting. This information is essential prior to initiating a wider roll out of the communication guide in primary dental care. The current study aimed to obtain this information by investigating the extent to which the oral cancer communication guide is implemented by the dental team (intervention fidelity delivery) and its impact on the dental patient (intervention fidelity receipt).^27^ Specifically, the following research questions were addressed:

1. How do patients respond to discussions about oral cancer in the dental practice? (fidelity receipt)
  - Does talking about oral cancer raise anxiety?
  - What questions do patients ask?
2. How is the communication guide used in everyday practice? (fidelity delivery)
  - Which topics of the communication guide are included in discussions about oral cancer?
  - How long do discussions about oral cancer take?
  - Do patient characteristics influence the extent to which a dentist talks to a patient about oral cancer?

## 2 METHODS

### 2.1 Study design

This observational study involved analysis of audio recordings from routine dental consultations in primary care linked with patient questionnaires completed before and after their appointment. Observation is the gold standard methodology to assess intervention fidelity delivery.^27^ Adding patient report to this allows additional insight into fidelity receipt.^28^

### 2.2 Participants

Participants were recruited from one primary care dental practice in South‐East London, England, UK, under the care of one primary care dentist who was familiar with the communication guide through a condensed session of the training described by Awojobi et al, (2016).^23^ The practice treats both NHS and private patients, but only NHS patients were invited to take part in this study. Inclusion criteria for the study were: English‐speaking patients over 18 years of age who had a routine NHS dental check‐up (either as a new or existing patient) during the study period (6 weeks during August–September 2019).

### 2.3 Procedure

All patients who had booked a check‐up appointment during the study period (consecutive case sampling) were given a letter signed by the dentist and a detailed information sheet about the study. This was either mailed to their home address or given to them at the practice if they were booking an appointment in person. On the day of the appointment, the researcher approached the patient to discuss the study. Those wishing to take part were asked to provide written informed consent and then completed a pre‐appointment questionnaire in the waiting room. The appointment with the dentist was audio‐recorded using a digital recorder (Philips, DVT 4010). After the dental appointment, the dentist and patient reconfirmed their consent for use of the audio recording and participants completed a post‐appointment questionnaire.

The participants were initially told (in the information sheet) that the focus of the research was on ‘communication and anxiety’ rather than specifically about ‘communication *about oral cancer* and anxiety’. This was because raising the topic in advance could have affected the communication occurring in the consultation (e.g., the patient may ask the dentist about oral cancer, which they would not have normally done) thus affecting the results. Following the British Psychological Society's Code of Human Research Ethics, the researcher debriefed each participant following completion of the post‐appointment questionnaire and asked to confirm if they were still willing for their data to be included in the study. An information leaflet about oral cancer was provided to those who wanted further information. The study procedure received HRA approval as part of a larger study assessing the impact of training on dental practitioners (REF: 19/NE/0221; IRAS project ID:246499).

### 2.4 Measures

#### 2.4.1 Questionnaires

The pre‐appointment questionnaire collected data on patients' state anxiety measured by the 6‐item Spielberger's State Trait Anxiety Indicator (STAI‐6).^29^ The scale has been shown to be valid and reliable in a range of samples and is a commonly used measure of anxiety that distinguishes between anxiety as a general aspect of personality (trait anxiety) and anxiety as a response to a specific situation (state anxiety). The STAI has been shown to be sensitive to change in anxiety even over a short duration.^29^, ^30^, ^31^ Scores range from 6 to 24 with higher scores indicating higher anxiety. For two patients with missing data (each missed one item), scores were pro‐rated. The Cronbach alpha coefficient was 0.67 for the STAI‐6 pre‐appointment and 0.75 for the STAI‐6 post‐appointment.

The pre‐appointment questionnaire also measured dental anxiety (via the Modified Dental Anxiety Scale [MDAS]),^32^ current level of pain (no pain, some pain, considerable pain or pain which could not be more severe), socio‐demographic details (age, sex, NHS status), medical history, alcohol consumption using the 3‐item Alcohol Use Disorders Identification Test Consumption (AUDIT‐C)^33^, ^34^ and tobacco use. The post‐appointment questionnaire collected current level of pain in addition to the STAI‐6. All questionnaires were piloted for face validity and ease of comprehension by five dental patients at a dental hospital.

### 2.5 Analysis

#### 2.5.1 Audio‐recordings

Topics discussed: Two researchers (GB, KS) independently listened to each audio recording to determine which of the topics in the communication guide were discussed in each consultation. The researchers used a checklist of the 10 topics with clear definitions for each topic. Each researcher indicated whether the topic was or was not included in the consultation. Overall, there was 95% agreement between the two raters across the 10 topics. For the 5% where there was disagreement, a third researcher (SES) rated the verbatim transcript to reach final agreement. The agreed ratings were used to generate a measure of number of topics discussed (range 0–10) for each participant.

Duration: Two researchers (GB, SES) listened to each audio recording to determine the duration of each discussion about oral cancer (measured in seconds) within each appointment.

Patient responses: One researcher (RE) analysed the anonymized verbatim transcripts of the audio recordings to monitor patient's verbal responses to discussions about oral cancer. Using deductive thematic coding, the researcher determined if patient responses to the initial discussion about oral cancer were positive, negative or neutral. In addition, all patient questions in response to the discussion about oral cancer were identified. In acknowledging the coding of responses is subjective, the initial coding was then checked and confirmed by a second researcher (SES) to ensure credibility of the analysis.

#### 2.5.2 Statistical analysis

Statistical data analysis was conducted using SPSS version 27. KS‐Lilliefors tests indicated non‐normal distribution of STAI‐6, the number of topics discussed and duration of discussions, therefore non‐parametric tests were used in analyses. A Wilcoxon Signed test was used to evaluate differences in STAI‐6 scores before and after the appointment. Based on the effect size of 0.3 (medium), alpha of 0.05 and a power to detect differences at 80%, a sample size of 82 participants was required to detect differences in anxiety over time.

Spearman rank correlation coefficients were calculated as a measure of the association between levels of state anxiety after the consultation and the number of topics discussed. Spearman rank correlation coefficients were also used to determine associations between patient characteristics and number of topics discussed, and state anxiety after the appointment. Due to multiple correlation calculations, statistical significance was considered at *p* < .01 to protect from type 1 error.

## 3 RESULTS

### 3.1 Participant characteristics

One hundred and six patients were eligible for inclusion during the six weeks study period. Of these, six did not attend. Consent to take part in the study (from both patient and dentist) was obtained for 80 patients. Adequate audio recordings were acquired for 77 (96%) of these participants. No participants withdrew their data after the debriefing. Table 1 summarizes the characteristics of the participants. The age of the participants ranged from 18 to 89 years (mean = 49.1 years; SD = 21.0). Most participants (*n* = 71, 92%) were paying for their NHS dental treatment. The remainder were either exempt due to pregnancy (*n* = 3, 4%) or being in receipt of income benefits (*n* = 3, 4%). Current tobacco use was reported by 7 participants (9%). Twenty participants (26%) reported former tobacco use and 52 (65%) participants had never smoked. Eleven participants (14%) did not consume alcohol. Twenty‐two participants (29%) had an alcohol intake indicative of risk of dependence as measured by the AUDIT‐C (mean Audit‐C score = 3.2; SD = 2.0). Scores on the Modified Dental Anxiety Scale ranged from 5 to 23 (mean = 11.1; SD 4.0). The majority (*n* = 45, 58%) were moderately anxious and 3 (4%) were very anxious, with levels akin with dental phobia.

**TABLE 1:** Participant characteristics (*N* = 77)

Variable	*N*	%	M (SD)	Median
Age				
18–39 yrs	32	42	49.1 (21.0)	51.0
40+ yrs	45	58		
Sex				
Female	41	53	‐	‐
Male	36	47		
Alcohol (AUDIT‐C)				
No alcohol consumption	11	14	3.2 (2.0)	3.0
Lower risk of alcohol dependence	44	57		
Higher risk of alcohol dependence	22	29		
Tobacco use				
Never smoked	50	65	‐	‐
Used to smoke	20	26		
Currently smoke	7	9		
Pain before appointment				
No pain	64	83	‐	‐
Some pain	8	10		
Considerable pain	5	7		
Pain after appointment				
No pain	66	86	‐	‐
Some pain	8	10		
Considerable pain	3	4		
Dental Anxiety				
Not dentally anxious (MDAS 5–9)	29	38	11.1(4.0)	11.0
Fairly dentally anxious (MDAS 10–18)	45	58		
Very dentally anxious (MDAS 19+)	3	4		
State anxiety (STAI‐6) before appointment	‐	‐	9.1 (2.6)	9.0
State anxiety (STAI‐6) after appointment	‐	‐	8.1 (2.8)	7.0
Medical history				
No comorbidities	45	58	‐	‐
Anxiety or depression	13	17		
Gastrointestinal condition	12	16		
Joint or bone condition	8	10		
Heart disease	5	7		
Cancer	4	5		
Lung disease	4	5		
Diabetes	4	5		

### 3.2 Talking about oral cancer

All consultations included a discussion about oral cancer to some extent. The dentist spoke to patients about oral cancer for between 34 and 193 s (mean = 95.2 s; SD = 34.5 s; median = 93.5 s). Sometimes, part of this discussion was during the intraoral examination (e.g. “*So now I'm just checking in your mouth, the soft tissues for any lumps and bumps and red patches, white patches. And that looks … that's good”* [P04]).

On average, the dentist discussed seven different topics with each patient when talking about oral cancer (Mean = 7.1 [SD = 0.9]; Median = 7.0; Range: 3–8 out of possible 10). Table 2 displays the extent to which each topic was discussed with patients. The dentist informed each patient that they were being screened for oral cancer, specifically using the word ‘cancer’. They also informed each patient of the main signs and symptoms of oral cancer. In the vast majority of appointments, the dentist also informed the patient about where in the mouth oral cancer can develop, the risk factors for oral cancer, and offered reassurance after the oral cancer screen (e.g. “*So you've passed your cancer screening*” [P068]).

**TABLE 2:** Frequency with which each topic of the communication guide was discussed

Topic		Consultations in which topic was discussed	
		*N*	(%)
A	Actually say ‘cancer’	77	100
	Reassurance [or referral] after screening	72	94
B	Asking about patient's prior knowledge	65	84
	Sign and symptoms of oral cancer	77	100
C	Three‐week rule for symptoms	68	88
	Where to seek help for symptoms	1	1
D	Where oral cancer can develop	76	99
	Risk / Risk factors for oral cancer	73	95
	Finding mouth cancer early saves lives	41	53
E	Barriers to seeking help	0	0

Red, Yellow, Green, Purple, Blue are encouraging early diagnosis of mouth cancer in general dental practice.

The dentist varied in the extent to which they encouraged the conversation to be interactive, with 84% of patients being asked what they knew prior to a discussion of signs and symptoms. Specific advice on the duration of symptoms (three‐week rule) was included in 88% of consultations. Topics that were rarely discussed were ‘where to seek help for symptoms’ (1%) and ‘barriers to seeking help’ (0%).

### 3.3 Talking about oral cancer and patient anxiety

The average level of state anxiety was lower after the appointment (median STAI‐6 = 7.0) compared to before the appointment (median STAI‐6 = 9.0; z = −2.83, *p* < .01).

There was no correlation between the number of oral cancer topics discussed and patient state anxiety after the appointment (Spearman's Rho = 0.13, *p* = .128) or between the number of oral cancer topics discussed and the change in state anxiety pre‐ to post‐appointment (Spearman's Rho = 0.02, *p* = .422, *N* = 74).

Table 3 summarizes associations between patient characteristics and number of topics discussed and state anxiety after the appointment. Smoking status was the only factor associated with the number of oral cancer topics discussed (Spearman's Rho = 0.27, *p* < .01). The dentist discussed more oral cancer topics with patients who smoked or used to smoke. The dentist discussed fewer oral cancer topics with patients who had a history of cancer, and more topics with those who had history of anxiety or depression, but these associations were only significant at the *p* < .05 level (Spearman's Rho = 0.21, *p* < .05).

**TABLE 3:** Factors associated with number of topics discussed and state anxiety

Variable	Association with number of topics discussed		Association with state anxiety (STAI‐6) after the appointment	
	Spearman's Rho	*p*	Spearman's Rho	*p*
Age (yrs)	−0.05	.333	−0.07	.281
Sex^a^	0.10	.195	0.11	.171
Alcohol consumption (AUDIT‐C score)	0.12	.152	0.17	.066
Smoking^a^	0.27	<.01	0.10	.190
Pain before appointment^a^	−0.05	.345	0.20	.041
Medical History^a^				
Heart disease	0.02	.443	−0.05	.348
Cancer	−0.21	.032	0.11	.169
Lung disease	0.17	.067	−0.05	.341
Joint or bone condition	−0.04	.371	0.02	.444
Gastrointestinal condition	−0.17	.074	−0.10	.197
Diabetes	0.004	.485	−0.08	.238
Anxiety or Depression	0.21	.035	−0.02	.426
Dental anxiety (MDAS)	0.001	.498	0.21	.034
State anxiety (STAI‐6) before appointment	0.08	.246	0.31	<.01
State anxiety (STAI‐6) after appointment	0.13	.128	‐	‐

^a^ Categorical variables were dichotomized as follows for analysis: Sex: 0 = female; 1 = male Smoking: 0 = never smoked; 1 = currently smoke or used to smoke Pain before appointment: 0 = no pain; 1 = some or considerable pain medical history: 0 = no history of condition; 1 = presence of condition.

State anxiety before the appointment was associated with state anxiety after the appointment (Spearman's Rho = 0.31, *p* < .01). Those who were more anxious prior to the appointment were more anxious after the appointment. Dental anxiety was associated with state anxiety after the appointment, but not at the *p* < .01 level (Spearman's Rho = 0.21, *p* < .05).

### 3.4 Patient responses to talking about oral cancer

Patient's initial responses to hearing about oral cancer screening were either short neutral replies (*n* = 39, 51%) such as “okay”, “fine,” “right” or positive comments (*n* = 17, 22%) such as “*My goodness dentistry has really come on*” [P008]; “*Okay. That's brilliant*” [P014]; “*Sure, that sounds good*.” [P028]. The remaining 22 patients (27%) gave no audible response. No patients reacted negatively to the discussion.

Patient questions were unprompted rather than invited by the dentist. There were relatively few (*n* = 8) patient questions in response to talking about oral cancer screening. Those that were asked related to either the novelty of screening for oral cancer (*n* = 4) e.g. “*Is it something new?*” [P046]; their own symptoms (*n* = 3), for example, “*I've got sore bit just here, um and there*” [P104]; or risk factors for oral cancer (*n* = 1), for example, “*What is the age that's vulnerable for mouth cancer?*” [P029].

## 4 DISCUSSION

A key objective of this study was to investigate how patients respond to conversations about oral cancer in routine dental consultations. To our knowledge, this is the first study that has done so, and one of the few studies that has collected enriched data on dentist–patient communication by audio‐recording routine dental appointments in general dental practice. Reassuringly, conversations about oral cancer did not appear to have impact on the level of patient anxiety. Rather than discussion about oral cancer, it was state anxiety before the appointment that was associated with state anxiety after the appointment: those patients who were anxious to begin with were more likely to be anxious after the appointment. Patients with risk factors for cancer (e.g., smoking, alcohol, advancing age) had similar levels of anxiety to those without risk factors, even after a discussion about oral cancer took place, although we are mindful that there were only a small number of patients who currently smoked in the sample. State anxiety levels were lower after the appointment compared with prior to the appointment. This does not necessarily mean that the oral cancer discussion led to the decrease in anxiety. However, it is reasonable to conclude that when the dentist talked about oral cancer it did not lead to an increase in anxiety. Patients were accepting of the discussion about oral cancer, with many commenting that it was a good initiative. This supports previous findings that dental patients want to be informed^20^ and that cancer screening and awareness interventions may result in lower levels of anxiety.^22^, ^35^, ^36^

Dentists have previously raised concerns that they may not have time or sufficient knowledge to answer patient questions that are raised following a discussion about oral cancer.^18^ However, this study indicates that patient questions are infrequent, and on topics that can be answered without problem. In addition, the discussion about oral cancer can be combined with the oral cancer screening, thus limiting the impact of adding a discussion about oral cancer to already time‐limited consultations.

Audio‐recording the consultations enabled insight into how the dentist spoke about oral cancer as well as the patients' reactions, enabling assessment of fidelity delivery and receipt. The dentist included many of the communication guide topics apart from encouraging help‐seeking should patients notice symptoms in the future—either in terms of whom to seek help from or ways to overcome barriers to seek help. Training sessions in the use of the communication guide should consider this as an area of focus. This study found that the dentist adjusted the discussion about oral cancer in light of a patient's smoking status, with a more extensive discussion with those who have or used to smoke, as recommended in the communication guide. No other patient characteristics appeared to influence the extent to which the dentist talked to patient about oral cancer, indicating equity in implementation of the intervention.

The main limitation of the study is that the findings are restricted to one dentist who may not be representative of others, whom may have different level of experience, communication skills or practice settings. Other limitations include lack of a control group for whom oral cancer was not discussed, and absence of data on additional patient characteristics (e.g. socio‐economic status, ethnicity) that may have influenced outcome measures. The sample size was also five participants short of that required for statistical power to detect differences. It may not be possible to generalize these results to other patients as the sample included a higher number of participants who reported moderate levels of dental anxiety (58%) compared with general population (36%) but fewer (4%) who reported being very dentally anxious compared with general population (12%).^37^ The majority of the sample were patients with relatively low risk of oral cancer. This is not only a consideration for the results of this study but also the potential efficacy of the intervention, given those at high risk of oral cancer may not regularly attend the dentist. Further, as the focus of the analysis of audio recordings was on the topics of the dentist–patient communication about oral cancer, additional analysis focusing on the quality of communication may be useful. For instance, use of non‐verbal and paralinguistic communication and the extent to which the dentist made information provision personally relevant to engage the patient and aid memory.

Whilst recognizing these limitations, especially with regard to generalizability of the results, the study has demonstrated that the dentist was able to discuss oral cancer within the confines of a time‐pressured appointment and this did not raise patients' anxiety. The findings indicate that using an evidence‐based guide to talk about oral cancer does not appear to raise patients' anxiety and this should encourage endeavours to raise awareness of oral cancer within the dental practice. Future work could assess the effectiveness of the intervention on raising cancer awareness, including duration of impact. Wider implementation will require development of accessible training resources that support and motivate the dental team as well as ensuring practitioners have the skills and beliefs in their capabilities to incorporate talking about oral cancer into their daily practice. Of course, there are additional hurdles to encouraging early diagnosis within the dental setting, not least dental attendance and ensuring routine oral cancer screening for all adult patients. This is especially the case following the COVID‐19 pandemic where access to dentistry has been limited and inequalities exacerbated.^38^, ^39^

## FUNDING INFORMATION

This research was funded by an Oral Health Innovations (OHI PreViser Award) Research grant, administered through the Oral & Dental Research Trust.

## CONFLICT OF INTEREST

None.

## Data Availability

The data that support the findings of this study are available on request from the corresponding author. The data are not publicly available due to privacy or ethical restrictions.
